# Supplementary material for: Environmental DNA provides higher resolution assessment of riverine biodiversity and ecosystem function via spatio-temporal nestedness and turnover partitioning
Source: Commun Biol. 2021 May 3;4:512. doi: 10.1038/s42003-021-02031-2 (PMC8093236; doi:10.1038/s42003-021-02031-2)
Supplement: Supplementary file 1 — Reporting Summary [file 42003_2021_2031_MOESM1_ESM.pdf]

nature research

Corresponding author(s): Mathew Seymour

Last updated by author(s): Mar 15, 2021

Reporting Summary

Nature Research wishes to improve the reproducibility of the work that we publish. This form provides structure for consistency and transparency in reporting. For further information on Nature Research policies, see our Editorial Policies and the Editorial Policy Checklist.

Statistics

For all statistical analyses, confirm that the following items are present in the figure legend, table legend, main text, or Methods section.

n/a	Confirmed	
☐	☒	The exact sample size (*n*) for each experimental group/condition, given as a discrete number and unit of measurement
☐	☒	A statement on whether measurements were taken from distinct samples or whether the same sample was measured repeatedly
☐	☒	The statistical test(s) used AND whether they are one- or two-sided<br>*Only common tests should be described solely by name; describe more complex techniques in the Methods section.*
☐	☒	A description of all covariates tested
☐	☒	A description of any assumptions or corrections, such as tests of normality and adjustment for multiple comparisons
☐	☒	A full description of the statistical parameters including central tendency (e.g. means) or other basic estimates (e.g. regression coefficient) AND variation (e.g. standard deviation) or associated estimates of uncertainty (e.g. confidence intervals)
☐	☒	For null hypothesis testing, the test statistic (e.g. *F*, *t*, *r*) with confidence intervals, effect sizes, degrees of freedom and *P* value noted<br>*Give P values as exact values whenever suitable.*
☒	☐	For Bayesian analysis, information on the choice of priors and Markov chain Monte Carlo settings
☒	☐	For hierarchical and complex designs, identification of the appropriate level for tests and full reporting of outcomes
☒	☐	Estimates of effect sizes (e.g. Cohen's *d*, Pearson's *r*), indicating how they were calculated

Our web collection on statistics for biologists contains articles on many of the points above.

Software and code

Policy information about availability of computer code

Data collection	Software was not used for data collection
Data analysis	R was used for analyses with standard coding and command functions

For manuscripts utilizing custom algorithms or software that are central to the research but not yet described in published literature, software must be made available to editors and reviewers. We strongly encourage code deposition in a community repository (e.g. GitHub). See the Nature Research guidelines for submitting code & software for further information.

Data

Policy information about availability of data

All manuscripts must include a data availability statement. This statement should provide the following information, where applicable:

- Accession codes, unique identifiers, or web links for publicly available datasets
- A list of figures that have associated raw data
- A description of any restrictions on data availability

Data including amplicon sequence data, ASV matrices and environmental data associated with the manuscript will be deposited on figshare. doi 10.6084/m9.figshare.14159579.v1

Field-specific reporting

Ecological, evolutionary & environmental sciences study design

All studies must disclose on these points even when the disclosure is negative.

Study description	We investigated the effects of landuse and seasonality on headwater community richness and functional diversity, to assess spatio-temporal dynamics between eDNA and traditional methods. Headwater riverine biodiversity is one of the longest standing realms of ecology and a key component of current freshwater biomonitoring and assessment. Using an experimental design which utilizes headwater sampling sites to associate local environmental conditions located within the same environmentally heterogeneous geographic region (e.g. catchment). We utilized a combined eDNA and traditional based biodiversity assessment approach to allow for direct comparison of historically supported ecological expectations from the traditional methods with molecular based eDNA methods.
Research sample	Sampling included traditional freshwater macroinvertebrate sample following established biomonitoring practices that are widely accepted and reproducible across the existing literature. We also compared with eDNA based assessment, which is a growing field using the most current methods for determining macroinvertebrate diversity.
Sampling strategy	The study was created to specifically generate statistical tested groups by balancing each of the planned explanatory variables used in the study (season, landuse and sampling method).
Data collection	Data collection was primarily conducted by the lead author (MS), with assistance by co-author PS. All data related information was check and verified via MS and PS during the collection period. Subsequent data processing where other individuals were tasked with different aspects of the data were coordinated directly with MS.
Timing and spatial scale	The study utilized a regional spatial scale across an entire drainage in North Wales, with a seasonal temporal sampling over a year.
Data exclusions	All data was included in the study
Reproducibility	Reproducibility was assessed by the repeated sampling design for each level of the experiment, including between time points, land use type and sampling methods. Each of these levels were balanced and replicated to allow for direct comparisons.
Randomization	Samples for each time point were processed in a random manner, this included the order of the extraction and sequencing
Blinding	Blinding was naturally included in the experiment via the use of eDNA sampling, whereby the final data was not unknown until the final sequencing run was completed. The taxonomic samples were identified by individuals with no prior knowledge of the site locations.

Did the study involve field work? ☒ Yes ☐ No

Field work, collection and transport

Field conditions	Mild to cool conditions
Location	North Wales head water sites
Access & import/export	Sampling locations occurred within country with regards to the laboratory location. eDNA sampling was noninvasive and involved water sampling. Taxonomic sampling involved non-destructive kicknet sampling and does not require permits with regards to sampling macroinvertebrates
Disturbance	eDNA sampling involves zero disturbance. Kick-netting involves minuscule substrate disturbance to river beds by literally kicking a small area of the surface with your foot to sample insects and is a common practice for practitioners and hobbyist.

Reporting for specific materials, systems and methods

We require information from authors about some types of materials, experimental systems and methods used in many studies. Here, indicate whether each material, system or method listed is relevant to your study. If you are not sure if a list item applies to your research, read the appropriate section before selecting a response.

Materials & experimental systems

n/a	Involved in the study	
☒	☐	Antibodies
☒	☐	Eukaryotic cell lines
☒	☐	Palaeontology and archaeology
☒	☐	Animals and other organisms
☒	☐	Human research participants
☒	☐	Clinical data
☒	☐	Dual use research of concern

Methods

n/a	Involved in the study	
☒	☐	ChIP-seq
☒	☐	Flow cytometry
☒	☐	MRI-based neuroimaging
