# Supplementary material for: Environmental DNA provides higher resolution assessment of riverine biodiversity and ecosystem function via spatio-temporal nestedness and turnover partitioning
Source: Commun Biol. 2021 May 3;4:512. doi: 10.1038/s42003-021-02031-2 (PMC8093236; doi:10.1038/s42003-021-02031-2)

Environmental DNA provides higher resolution assessment of riverine biodiversity and ecosystem function via spatio-temporal nestedness and turnover partitioning

Mathew Seymour[1]*, François K. Edwards[2], Bernard J. Cosby[3], Iliana Bista[4,5], Peter M. Scarlett[2], Francesca L. Brailsford[6], Helen C. Glanville[6], Mark de Bruyn[7], Gary R. Carvalho[6], Simon Creer[6]

1. Department of Ecology, Swedish University of Agricultural Sciences, Uppsala, Sweden
2. Centre for Ecology &Hydrology, Wallingford, United Kingdom
3. Centre for Ecology & Hydrology, Environment Centre Wales, Bangor, United Kingdom
4. Department of Genetics, University of Cambridge, Cambridge, United Kingdom
5. Wellcome Sanger Institute, Hinxton, United Kingdom
6. School of Natural Sciences, Bangor University, Bangor, United Kingdom
7. The University of Sydney, School of Life and Environmental Sciences, Sydney, Australia

Corresponding author; *mathew.seymour@slu.se

Supplementary Figure 1. Visual illustration of the environmental PCA used to construct the environmental gradient (PC1). The colors indicate the landuse type of the respective points and correspond to the associated colors in Figures 1 and 2, with black = urban, green = agriculture, blue = forest, red = acid grasslands and brown = moorlands. Polygons are used to indicate landuse groups and are colored in the same manner as the points themselves.

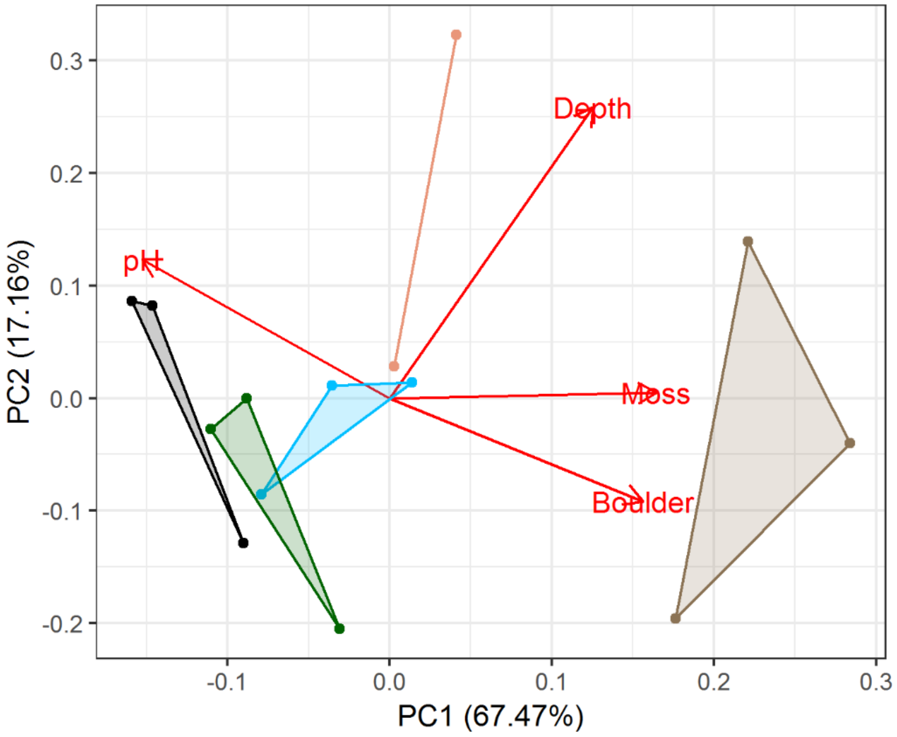

Supplement: Supplementary file 2 — Supplementary Information [file 42003_2021_2031_MOESM2_ESM.pdf]
