# Supplementary material for: Environmental DNA provides higher resolution assessment of riverine biodiversity and ecosystem function via spatio-temporal nestedness and turnover partitioning
Source: Commun Biol. 2021 May 3;4:512. doi: 10.1038/s42003-021-02031-2 (PMC8093236; doi:10.1038/s42003-021-02031-2)
Supplement: Supplementary file 4 — Description of Additional Supplementary Files [file 42003_2021_2031_MOESM4_ESM.docx]

**File name**: Supplementary Data 1

**File Description:** Breakdown of genera per landuse type given as percent of total genera richness per site, by method.
